# Library of the American Medical Association, Washington, D. C.

**Published:** 1869-08

**Authors:** Robert Reyburn, Joseph M. Toner

**Affiliations:** Librarian; Library Com.


### Library of the American Medical Association, Washington, D. C.

The Medical Profession, and Scholars generally, are aware of the ephemeral form in which most of the early American contributions to the literature of medicine were given to the world, and, indeed, in which many of the more recent are being published. This condition of much of our professional literature is deeply regretted by all, and particularly by those whose taste and research lead them to refer to this class of works, when the fact is made apparent that whole editions of tracts and books have entirely perished through neglect. With a view to provide against such a contingency, and preserve, for the benefit of the profession, in some accessible and central locality, copies of all home medical publications, the American Medical Association, at its annual meeting in May last, resolved to establish at Washington, D.C., a Library or Repository of American medical works, to which it is believed all the current medical literature of our country will be cheerfully, promptly and constantly contributed.

It is designed that this repository shall contain copies of every contribution by American physicians to the literature and science of medicine, from the earliest settlement of our country, no matter how or where published, including all the books, pamphlets, journals, and even unpublished manuscripts, that can be collected.

Nearly all physicians have some book or pamphlet of the character indicated, which may contain facts relative to the Diseases of his section published no where else, which they can contribute without inconvenience, and which of itself is of trifling value, yet when many such treatises are assembled together from all parts of our country, embracing its nosology from the earliest period of its settlement, they will form a collection of the greatest importance to the profession.

The Librarian of Congress has kindly consented to receive and preserve as a special deposit, in the Government fire-proof building, any collection of medical works the American Medical Association may make, and will keep them in condition to be readily consulted. The accommodation thus offered the Association for accumulating and preserving its library free of cost is generous and most encouraging. Gentlemen having scarce and valuable American medical publications will not hesitate to deposit them in such a safe, central and national repository, where they will be preserved from destruction and their usefulness secured to the profession.

An appeal for contributions to this library is now made, personally and distinctly, to each and every American Physician, Medical Publisher, and Editor, to deposit copies of their works in this repository, where they will be carefully kept for reference and catalogued with the name of the donor.

We, the undersigned, members of the American Medical Association, having been selected to carry into effect, as far as practicable, the resolution of the Association to establish a Library, have now completed all the necessary arrangements for the reception and preservation of those books which may be sent to our care. Contributions of the class of works mentioned, are therefore respectfully and earnestly solicited from every source. Packages may be sent by mail or by Adams' express, to either of us, which will be promptly acknowledged on reception, and a record of titles kept. The library mark of the Association will be pasted on the inside of the cover of each Volume, which will contain also the name of the donor.

Hoping that you may further the project to the extent of adding at least your own productions,

We remain, respectfully,

ROBERT REYBURN, M. D., Librarian,
JOSEPH M. TONER, M. D., Library Com.

